# Temporal dynamics of muscle strength recovery following acute cold-water immersion: a systematic review and meta-analysis

**DOI:** 10.7717/peerj.21537

**Published:** 2026-07-16

**Authors:** Yang Zhu, Lele Yang, Tao Liu, Fuya Yao, Qilong Wang, Zheng Yi

**Affiliations:** Institute of Physical Education and Training, Capital University of Physical Education and Sports, Beijing, China

**Keywords:** Cold water immersion, Muscle damage, Muscle strength, Delayed onset muscle soreness, Explosive power

## Abstract

**Objective:**

This study aimed to investigate the time-dependent effects of a single bout of acute cold-water immersion (CWI) on the recovery of post-exercise maximal muscle strength, explosive power, biochemical markers of muscle damage, and subjective pain, with the specific goal of determining the optimal temporal window for its practical application.

**Methods:**

A systematic search was conducted in PubMed, Web of Science, Cochrane Library, and Embase databases for studies published from inception to September 1, 2025. Randomized controlled trials (RCTs) comparing a single bout of acute CWI with passive recovery regarding their effects on post-exercise strength recovery in healthy individuals were included. Methodological quality, risk of bias, and certainty of evidence were assessed using the Physiotherapy Evidence Database (PEDro) scale, the Risk of Bias 2 (RoB 2) tool, and the Grading of Recommendations Assessment, Development and Evaluation (GRADE) approach, respectively. Statistical analysis was performed using Stata-MP 18.0 software.

**Results:**

Twenty-two RCTs were included. For maximal voluntary isometric contraction (MVIC), no significant overall or time-dependent effects were observed (g = 0.08, 95% confidence interval (CI) [−0.11–0.26], *p* = 0.42; moderate certainty). For countermovement jump (CMJ) , the overall effect was not significant (g = 0.01, 95% CI [−0.25–0.28], *p* = 0.92), but a significant subgroup difference across time points was detected (*p* < 0.01; low certainty), with a transient impairment immediately post-exercise (0 h: g = −0.68, 95% CI [−1.16 to −0.20], *p* = 0.01) that was no longer evident by 24-48 h. For visual analog scale (VAS), CWI was associated with lower overall soreness scores (g = −0.58, 95% CI [−0.99 to −0.16], *p* = 0.01), although heterogeneity was substantial and certainty was very low. For creatine kinase (CK), the initial pooled effect suggested a small reduction (g = −0.43, 95% CI [−0.67 to −0.19], *p* < 0.01; low certainty), but this effect was attenuated to non-significance after trim-and-fill adjustment (g = −0.16, 95% CI [−0.44–0.12]). Cluster-robust sensitivity analyses preserved the direction of CK and VAS effects but attenuated their statistical significance.

**Conclusion:**

Acute CWI exerts outcome-specific effects on post-exercise recovery. It showed no meaningful effect on MVIC, transiently impaired immediate explosive performance, and may reduce subjective soreness, whereas the apparent CK benefit was not robust after adjustment for publication bias. The CK and VAS findings should be interpreted cautiously because heterogeneity, publication bias, and dependency-adjusted sensitivity analyses weakened the certainty of inference. Acute CWI may be most relevant when short-term symptom control and next-day recovery readiness are prioritized, but its timing should be considered carefully when another power-dependent task is scheduled soon after exercise.

## Introduction

In modern competitive sport, the time available for recovery between training sessions and matches is often limited by congested schedules and high training frequency (Page et al., 2023). High-intensity or unaccustomed exercise, especially exercise with a large eccentric component, can induce exercise-induced muscle damage (EIMD) (Proske & Morgan, 2001). Typical signs include a decline in force output, impaired jumping and sprint performance, increased delayed onset muscle soreness (DOMS), and elevated circulating markers of muscle damage (Crowther et al., 2017; Wei, Liu & Wang, 2025). If recovery is incomplete, the neuromuscular deficits caused by the previous load may persist into the next training session or competition. This can impair sport-specific performance, reduce training quality, and increase injury risk (Moore et al., 2022). Therefore, post-exercise recovery should not be viewed only as a supportive method for symptom relief. It should be considered a central part of load management and competition preparation (Mujika et al., 2018). Among the many recovery outcomes, strength recovery holds a central position (Suchomel, Nimphius & Stone, 2016). Muscle strength is the basis of athletic performance, movement control, and injury prevention. It is also a key indicator for training periodization and load management (Kellmann et al., 2018).

Cold-water immersion (CWI) has long been regarded as a representative recovery intervention in elite sport because it is easy to implement and requires little equipment (Versey, Halson & Dawson, 2013). Its main mechanisms include the effects of cold exposure and hydrostatic pressure. These effects promote vasoconstriction and fluid redistribution, reduce inflammatory responses and tissue swelling, and alter neuromuscular conduction and sensory input. Together, these responses may attenuate the symptoms of EIMD and accelerate recovery (Bleakley & Davison, 2009). However, although CWI is widely used in post-exercise recovery, its true effect on muscle strength recovery remains controversial (Leeder et al., 2011). Moore et al. (2022) found in a systematic review and meta-analysis that CWI is an effective recovery tool following high-intensity exercise, with positive effects on muscle strength, muscle soreness, CK levels, and perceived recovery 24 h post-exercise. However, following eccentric exercise, CWI showed a positive effect on muscle strength only at 24 h post-exercise. A randomized controlled trial by Garcia, Da Mota & Marocolo (2016) also showed that CWI negatively impacts performance in the short term, indirectly suggesting a time-dependent effect of CWI. This suggests that the efficacy of CWI may change across the recovery timeline rather than remain uniformly beneficial (Poppendieck et al, 2013). At the dosing level, Machado et al. (2015) provided early evidence on the combined role of water temperature and immersion duration. They suggested that 11–15 °C for 11–15 min was effective for short-term relief of DOMS. More recently, the network meta-analysis by Wang, Wang & Pan (2025) expanded the comparison across a wider range of dosing combinations and supported the selection of different temperature-duration protocols for different recovery goals. Even so, both the early dosing reviews and the recent network meta-analyses included multiple follow-up points, and the selected time points varied across studies. As a result, there is still no consistent and quantitative conclusion on the time-dependent effects of a single acute bout of CWI on muscle strength recovery at 0, 24, 48, and 72 h.

Additionally, attenuation of post-exercise inflammation is not uniformly beneficial, Recent evidence also indicates that prolonged or repeated application of CWI may exert unfavorable effects on muscle strength and hypertrophic adaptation (Roberts et al., 2015; Petersen & Fyfe, 2021; Piñero et al., 2024). Cytokine/redox signalling, satellite-cell activation and mechanistic target of rapamycin (mTOR)-mediated anabolic pathways are critical for repair and adaptation, CWI—while beneficial for short-term soreness relief and performance—may, if used routinely during adaptation-focused training, blunt the signals driving supercompensation (Broatch, Petersen & Bishop, 2018). Therefore, long-term use should balance short-term recovery goals with long-term adaptation goals. Furthermore, perceived recovery improvements may be partially influenced by expectation or placebo effects, which complicate interpretation of subjective outcomes (Broatch, Petersen & Bishop, 2014). In summary, the recovery effects of CWI may be influenced by various factors, including immersion temperature and duration, type and intensity of exercise, the training level of the subjects, and the timing of assessments (Anderson, Nunn & Tyler, 2018; Sánchez-Ureña, Rojas-Valverde & Gutiérrez-Vargas, 2018).

Finally, it is crucial to note that the recovery effects of CWI may exhibit a distinct time-dependent nature. Even when temperature, immersion duration, and site are consistent, CWI may exhibit different directions and intensities of effects at immediate (0 h), 24 h, 48 h, and 72 h post-exercise (Xiao et al., 2023). These time points were the most commonly reported in the literature and reflected typical short-term inter-session recovery windows. To comprehensively reflect the recovery of skeletal muscle following CWI, four complementary indicators are utilized in this study: maximal voluntary isometric contraction (MVIC), countermovement jump (CMJ), creatine kinase (CK), and visual analog scale (VAS) for pain. These indicators represent maximal strength, explosive power and neuromuscular function, biochemical markers of muscle damage, and subjective recovery experience, respectively (Afonso et al., 2021).Therefore, this study systematically reviews and conducts a meta-analysis to quantify the effects of acute CWI on strength-related indicators (MVIC, CMJ, CK, VAS) at different recovery time points, aiming to reveal its time-dependent characteristics and address the gaps in existing evidence, thereby providing more targeted, evidence-based recommendations for recovery strategies in training and competition contexts.

## Materials and Methods

### Registration

The study protocol was registered on PROSPERO (CRD420251145446) before data extraction. The systematic review and meta-analysis followed the Cochrane Handbook and PRISMA guidelines strictly (Page et al., 2021). This ensured transparency and consistency.

### Search strategy

**Figure 1:**
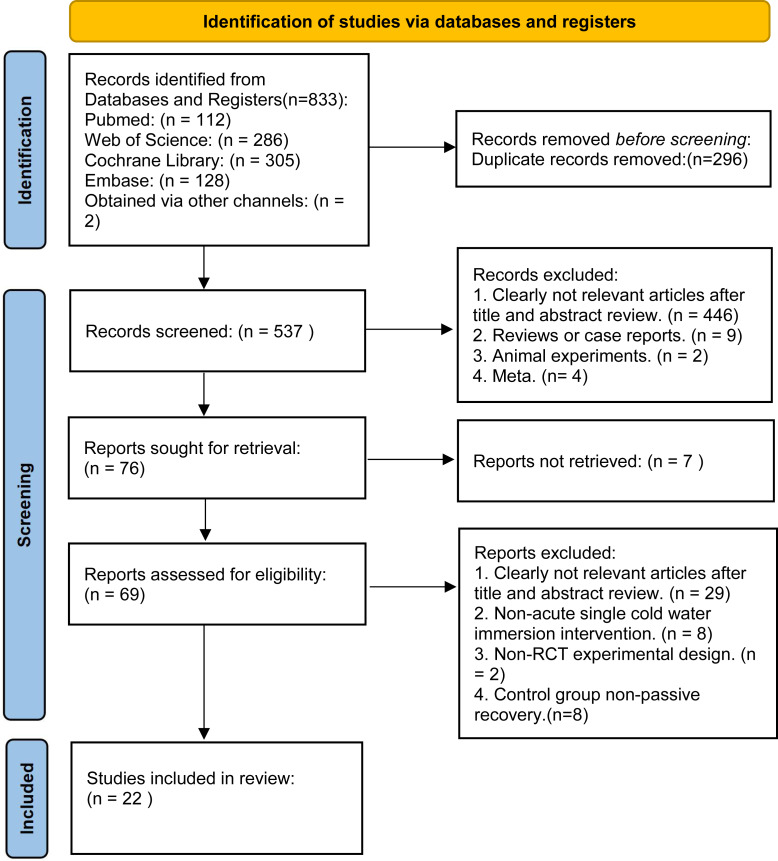
Flow diagram of the selection process.

**Figure 2:**
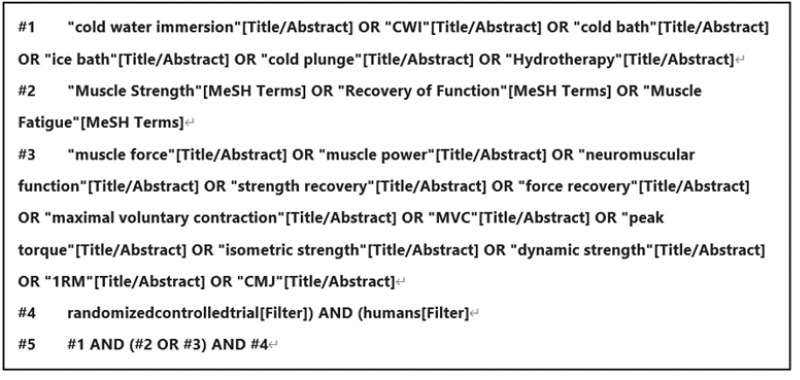
PubMed search formula.

Computerized searches were conducted in PubMed, Web of Science, Cochrane Library, and Embase databases, with the literature search period spanning from database inception to September 1, 2025. Additional literature was sought through other means to ensure comprehensive inclusion. The complete study-selection process is summarized in the PRISMA flow diagram (Fig. 1). The detailed PubMed search formula is provided in Fig. 2. The literature search strategy combined subject terms and free-text words, exemplified by the search strategy used in PubMed (Fig. 2). The full search strategies for all databases are provided Supplementary Material.

### Inclusion and exclusion criteria

Inclusion and exclusion criteria for the literature were established based on the Participants, Interventions, Comparisons, Outcomes, and Study Design (PICOS) principles (Arya, Kaji & Boermeester, 2021).

#### Inclusion criteria

(1) Conducted in healthy individuals, with no recent history of illness or chronic conditions.

(2) The acute CWI protocol must be administered within 1-hour post-exercise, and only once.

(3) The control group underwent seated passive recovery in thermometric conditions (20–25 °C).

(4) Baseline and post-intervention assessments must be conducted, with outcome measures including MVIC, CMJ, CK, and VAS. Monitoring time points must include at least one of the following: immediately post-CWI (0 h), 24 h, 48 h, or 72 h.

(5) Randomized controlled trials (RCTs), explicitly including both parallel-group designs and randomized crossover designs. For crossover trials, an adequate washout period between intervention periods must be implemented to ensure a complete return to baseline and minimize carry-over effects (Higgins et al., 2019).

#### Exclusion criteria

(1) Non-randomized trials, quasi-experimental designs, or randomized crossover trials that lack an adequate washout period (where the potential for carry-over effects cannot be reasonably excluded).

(2) Studies that include only CWI as the sole intervention; studies involving other recovery methods (*e.g.*, active recovery, stretching, massage) or nutritional supplements were excluded.

(3) Studies from which valid outcome data could not be extracted, and where clarification from the authors was not possible.

(4) Case reports, cohort studies, qualitative studies, systematic reviews or meta-analyses, study protocols, unpublished preprints, and conference abstracts.

(5) Duplicate publications.

### Literature screening

All retrieved citations were imported into EndNote X9, where an independent researcher (YZ) identified and removed duplicate records. Following this step, an initial screening of titles and abstracts was conducted independently by two reviewers (LL, TL) based on the predefined eligibility criteria. Irrelevant studies were discarded, and the full texts of potentially eligible articles were downloaded for in-depth assessment. Any disagreements between the two reviewers were settled through mutual discussion, with a third author (ZY) acting as an arbitrator when consensus could not be reached. The data extraction process was also performed and cross-validated by two independent reviewers, while a third investigator resolved any conflicting entries. Whenever data were found to be missing or unclear, we reached out to the corresponding authors of the original papers to acquire the necessary information.

### Data extraction

Data extraction was performed independently by two researchers utilizing a standardized Microsoft Excel template. The following parameters were extracted: 1. Basic information: first author, publication year, study type, and participant characteristics (sample size, gender, age, height, weight); 2. Study design: exercise type and protocol, CWI protocol, outcome measures, and monitoring time points.

### Quality assessment

The methodological quality and risk of bias of the included studies were independently evaluated by two reviewers. The revised Cochrane risk-of-bias tool for randomized trials (RoB 2) was employed to assess five key domains: the randomization process (Sterne et al., 2019), deviations from intended interventions, missing outcome data, measurement of the outcome, and selection of the reported result. Discrepancies were resolved through consensus or *via* arbitration by a third reviewer. Visual representations of the RoB 2 assessments were generated using the robvis web application (McGuinness & Higgins, 2020).

To provide a complementary quantitative evaluation of methodological rigor, the Physiotherapy Evidence Database (PEDro) scale was applied (De Morton, 2009). The PEDro scale comprises 11 items, with items 2–11 contributing to a maximum total score of 10. Studies scoring ≥ 6 were classified as having high methodological quality, 4–5 as moderate, and ≤ 3 as low. Finally, the overall certainty of evidence for the primary outcomes was appraised using the Grading of Recommendations Assessment, Development and Evaluation (GRADE) framework (Guyatt et al., 2008).

### Statistical analysis

All statistical analyses were performed using Stata-MP 18. To minimize confounding due to baseline differences, change scores (the difference between pre- and post-intervention means and their standard deviations) were used as effect size input. Effect size was measured using the standardized mean difference, with Hedges’ g applied to adjust for small sample bias. For multi-arm trials in which more than one CWI intervention arm shared a common control group, the relevant CWI arms were first combined within each outcome and time point before calculating the effect size, thereby avoiding double-counting of the shared control group (Axon, Dwan & Richardson, 2023). All combined effects were expressed as Hedges’ g and reported with corresponding 95% confidence intervals (CIs). Effect sizes were interpreted according to Cohen’s classification, divided into four categories: large effect (*g* > 0.8), medium effect (0.5 ≤ g ≤0.8), small effect (0.2 ≤ g <0.5), and negligible effect (g < 0.2) (Cohen, 2013). Descriptive statistics for continuous variables were presented as mean ± standard deviation (mean ± SD); statistical significance was set at a two-tailed *P* value < 0.05.

Heterogeneity was assessed using the *Q* test and *I*^2^ statistic: a fixed-effect model was used when *I*^2^ ≤ 50% and *P* ≥ 0.1, and a random-effects model was used when *I*^2^ > 50% or *P* < 0.1. The H^2^ statistic was additionally consulted to corroborate model selection when appropriate (Higgins et al., 2019). Time-dependent effects were examined using subgroup analyses at 0 h, 24 h, 48 h, and 72 h post-exercise, and subgroup differences were assessed using the test for subgroup differences (Qb). Sensitivity analysis was performed using the “leave-one-out” method (Tobias, 1999), which involved removing one study at a time and recalculating the pooled effect size to verify result stability. Because several studies contributed multiple effect sizes across time points or outcomes, an additional dependency-adjusted sensitivity analysis was conducted using inverse-variance weighted models with cluster-robust standard errors clustered by study ID (Cameron & Miller, 2015). This analysis was used to examine the robustness of the findings after accounting for within-study dependency and did not replace the primary random-effects models (Hedges, Tipton & Johnson, 2010). Publication bias was assessed visually using funnel plots, followed by Egger’s regression test for quantitative assessment (Egger et al., 1997). Where potential bias was identified, Duval and Tweedie’s “Trim and Fill” method was applied for correction (Duval & Tweedie, 2000).

## Results

### Studies search results

The initial database search yielded a total of 833 records (831 from databases and two from manual tracing). Following the removal of 296 duplicates using EndNote X9 software, the titles and abstracts of the remaining 537 records were screened, resulting in the exclusion of 461 articles. Subsequently, the remaining 76 full-text articles were retrieved and assessed for eligibility. Of these, 54 were excluded: 47 failed to meet the inclusion criteria, and seven had unavailable full texts. Ultimately, 22 studies met the eligibility criteria and were included in the quantitative synthesis (Sánchez-Ureña, Rojas-Valverde & Gutiérrez-Vargas, 2018; Amir, Hashim & Saha, 2017; Anderson, Nunn & Tyler, 2018; Broatch, Petersen & Bishop, 2014; Crowther et al., 2017; Dantas et al., 2019; Elias et al., 2012; Fonseca et al., 2016; Garcia, Da Mota & Marocolo, 2016; Li et al., 2023; Machado et al., 2016; Nasser et al., 2023; Pesenti et al., 2020; Richards et al., 2025; Rupp et al., 2012; Silva et al., 2018; Vaile et al., 2007; Wei, Liu & Wang, 2025; White, Rhind & Wells, 2014; Wiewelhove et al., 2018; Yoshimura et al., 2023; Takeda et al., 2014) (Fig. 1).

### Basic characteristics and information of included studies

A total of 22 studies were included in this review, published between 2008 and 2025. The total sample size of the included studies was 375 participants (362 males and 13 females), with the smallest sample size being seven participants (Pesenti et al., 2020) and the largest being 60 participants (Machado et al., 2016). The age range of participants was 15.6 to 48.9 years. Nine studies were randomized parallel-controlled trials (Sánchez-Ureña, Rojas-Valverde & Gutiérrez-Vargas, 2018; Amir, Hashim & Saha, 2017; Anderson, Nunn & Tyler, 2018; Dantas et al., 2019; Machado et al., 2016; Rupp et al., 2012; Wei, Liu & Wang, 2025; Wiewelhove et al., 2018; Yoshimura et al., 2023), and 13 were randomized crossover trials (Broatch, Petersen & Bishop, 2014; Crowther et al., 2017; Elias et al., 2012; Fonseca et al., 2016; Garcia, Da Mota & Marocolo, 2016; Li et al., 2023; Nasser et al., 2023; Pesenti et al., 2020; Richards et al., 2025; Silva et al., 2018; Vaile et al., 2007; White, Rhind & Wells, 2014; Takeda et al., 2014), all of which included washout periods. Nine studies involved healthy non-athlete participants (White, Rhind & Wells, 2014; Broatch, Petersen & Bishop, 2014; Machado et al., 2016; Amir, Hashim & Saha, 2017; Crowther et al., 2017; Sánchez-Ureña, Rojas-Valverde & Gutiérrez-Vargas, 2018; Yoshimura et al., 2023; Richards et al., 2025; Wei, Liu & Wang, 2025), and thirteen studies involved professional athletes (Anderson, Nunn & Tyler, 2018; Dantas et al., 2019; Elias et al., 2012; Fonseca et al., 2016; Garcia, Da Mota & Marocolo, 2016; Li et al., 2023; Nasser et al., 2023; Pesenti et al., 2020; Rupp et al., 2012; Silva et al., 2018; Vaile et al., 2007; Wiewelhove et al., 2018; Takeda et al., 2014). Exercise modalities included simulated competitions (Takeda et al., 2014; Garcia, Da Mota & Marocolo, 2016; Silva et al., 2018; Nasser et al., 2023; Li et al., 2023) (five studies), sport-specific training (Rupp et al., 2012; Elias et al., 2012; Fonseca et al., 2016) (three studies), high-intensity interval training (Sánchez-Ureña, Rojas-Valverde & Gutiérrez-Vargas, 2018; Anderson, Nunn & Tyler, 2018; Amir, Hashim & Saha, 2017; Broatch, Petersen & Bishop, 2014; Crowther et al., 2017; White, Rhind & Wells, 2014) (six studies), middle- and long-distance running (Wiewelhove et al., 2018; Dantas et al., 2019) (two studies), and eccentric or strength training (Vaile et al., 2007; Machado et al., 2016; Pesenti et al., 2020; Yoshimura et al., 2023; Richards et al., 2025; Wei, Liu & Wang, 2025) (six studies). No adverse reactions were reported in any of the studies. Specific information is provided in Tables 1 and 2.

**Table 1:** Basic information of the included studies.

**Included studies**	**Country of origin**	**Research type**	**Research participant**	**Age (years)**	**Sample size**		**Height (cm)/ Weight (kg)**
					**CWI**	**CON**	
**Vaile 2007**	AU	CS	Strength trainer	NR	12M	12M	NR
**Elias 2012**	AU	CS	Soccer players	20.9 ± 3.3	14M	14M	186.0 ± 7.2/79.6 ± 6.7
**Rupp 2012**	UK	PS	Soccer players 13M/9F	19.8 ± 1.1	12	10	174.0 ± 9.0/72.1 ± 9.1
**Broatch 2014**	AU	CS	Healthy male	24.0 ± 5.0	10M	10M	179.3 ± 6.6/78.7 ± 8.5
**Takeda 2014**	JP	CS	Rugby players	20.3 ± 0.6	20M	20M	174.0 ± 5.0/85.4 ± 2.0
**White 2014**	CA	CS	Healthy male	23.6 ± 3.7	8M	8M	180.8 ± 8.1/76.1 ± 8.6
**Garcia 2016**	BR	CS	Rugby players	23.0 ± 4.7	8M	8M	176.9 ± 4.5/87.5 ± 8.6
**Fonseca 2016**	BR	CS	Jiu-jitsu athlete	24.0 ± 3.6	8M	8M	NR/78.4 ± 2.4
**Amir 2017**	MY	PS	Healthy male	21.6 ± 2.3	8M	8M	167.2 ± 6.4/61.6 ± 11.1
**Anderson 2018**	UK	PS	Team event athletes	24.0 ± 2.0	9M	9M	178.0 ± 9.0/77.6 ± 14.2
**Crowther 2017**	AU	CS	Healthy male	27.0 ± 6.0	29M	29M	180.0 ± 8.0/80.0 ± 9.0
**Machado 2016**	BR	PS	Healthy male	20.8 ± 2.6	20M/20M	20M	174.0 ± 5.0/74.4 ± 11.2
**Silva 2018**	BR	CS	Jiu-jitsu athlete	21.8 ± 3.1	10M2F	10M2F	170.0 ± 5.0/72.1 ± 13.0
**Sánchez 2018**	CR	PS	Healthy male	21.8 ± 2.8	13M	13M	176.6 ± 5.3/73.2 ± 8.2
**Wiewelhove 2018**	DE	PS	Long-distance runner	30.5 ± 10.9	11M	12M	179.4 ± 6.2/75.5 ± 7.5
**Dantas 2019**	BR	PS	Male runners	31.6 ± 3.9	10M	10M	175.6 ± 6.3/77.7 ± 7.0
**Pesenti 2020**	BR	CS	Soccer players	16.5 ± 0.9	7M	7M	174.0 ± 5.2/69.2 ± 5.1
**Li 2023**	CN	CS	Basketball player	22.8 ± 0.8	10M	10M	179.0 ± 4.0/75.6 ± 6.6
**Nasser 2023**	TN	CS	Soccer players	21.1 ± 2.2	12M	12M	174.9 ± 4.6/72.4 ± 5.9
**Yoshimura 2023**	JP	PS	Healthy male	21.4 ± 0.8	8M	10M	171.7 ± 7.9/64.5 ± 7.6
**Richards 2025**	CA	CS	Healthy participants	23.3 ± 3.1	10M2F	10M2F	176.6 ± 8.1/84.1 ± 13.5
**Wei 2025**	CN	PS	Healthy male	21.1 ± 1.4	5M	5M	175.7 ± 4.5/69.5 ± 6.8

**Notes.**

CR Costa Rica MY Malaysia UK United Kingdom AU Australia BR Brazil LB Lebanon CH Switzerland KR South Korea CN China TN Tunisia CA Canada DE Germany JP Japan PS Randomized parallel-group trial CS Randomized crossover trial M Male F Female CWI Cold water immersion group CON Control group NR Not reported

**Table 2:** Interventional strategies and outcome indicators of the included studies.

**Included studies**	**Exercise protocol**	**Intervention program**		**Outcome indicator**	**Monitoring time point**
		**CWI**	**CON**		
**Vaile 2007**	Centrifugal Resistance Training	15.0 °C; 14 min; Submerged up to the shoulders	14 min PR	MVIC; CK	24;48;72
**Elias 2012**	Standardized Australian Football Training, HIIT	12.0 °C; 14 min; Immerse to the xiphoid process	14 min PR	CMJ; VAS	0;24;48
**Rupp 2012**	Yo-Yo Intermittent Recovery Test, HIIT	12.0 °C; 15 min; Soak up to the navel	15 min PR	CMJ	24;48
**Broatch 2014**	HIIT,4 sets × 30 s sprint, Rest interval: 4 min.	10.3 ± 0.2 °C; 15 min; Soak up to the navel	15 min PR	VAS	0;24;48
**Takeda 2014**	80- minute Rugby Simulation Training, HIIT	15.0 °C; 10 min; Full-body immersion	10 min PR	VAS; CMJ	24
**White 2014**	HIIT, 12 sets × 120 m sprint, Rest interval: 3 min.	10.0 °C; 10 min; Immersion to the iliac crest	10 min PR	VAS	0;24;48
**Garcia 2016**	HIIT Simulating Rugby Matches	8.9 ± 0.6 °C; 18 min; Immersion to the iliac crest	18 min PR	CMJ	0;24
**Fonseca 2016**	HIIT for Jiu-Jitsu Competition, 3 sets × 40 min	6.0 ± 0.5 °C; 16 min; Submerged up to the neck	16 min PR	CMJ	0;24;48
**Amir 2017**	10 sets of 10 repetitions CMJ	15.0 ± 1.0 °C; 15 min; Immersion to the iliac crest	15 min PR	CK; VAS	24;48;72
**Anderson 2018**	sprint-included interval training, 21 min interval running, Rest interval: 3 min.	C1:5.0 ± 1.0 °C; 12 min; Immersion to the iliac crest; C2:14.0 ± 1.0 °C, 12 min; Immersion to the iliac crest	12 min PR	CK	0;24;48;72
**Crowther 2017**	High-intensity interval training	15.0 °C; 14 min; Submerged up to the shoulders	14 min PR	VAS; CMJ	0;24;48
**Machado 2016**	75% of 1RM, 5 sets, eccentric resistance training	C1: 9.0 ± 1.0 °C; 15 min;Immersion to the iliac crest; C2: 14.0 ± 1.0 °C; 15 minImmersion to the iliac crest;	15 min PR	MVIC; CK; VAS	0;24;48;72
**Silva 2018**	HIIT for Jiu-Jitsu Competition, 5- min combat simulation (3- min interval)	12.0 °C; 6 min; Submerged up to the sternum	6 min PR	CK	0
**Sánchez 2018**	8 sets × 30-second maximum effort vertical jumps, 90 s rest between sets	12.0 ± 0.4 °C; 12 min; Soak up to the navel	12 min PR	CMJ	24;48
**Wiewelhove 2018**	Half Marathon (21.1 km)	15.0 °C; 15 min; Immersion to the iliac crest	15 min PR	CMJ; VAS; CK	0;24
**Dantas 2019**	10-kilometer run	10.0 °C; 10 min; Immersion to the anterior superior iliac spine	10 min PR	MVIC	0;24
**Pesenti 2020**	Two maximum knee extensions (60% of 1RM), 30 s rest between sets	10.0 °C; 10 min; Immersion to the iliac crest	10 min PR	MVIC	24;48;72
**Li 2023**	HIIT in Basketball Games	5.0 ± 1.0 °C; 12 min; Immersion to the iliac crest	12 min PR	CMJ	0;24
**Nasser 2023**	Simulated Soccer Match Shuttle Runs 75 min	11.3 ± 0.2 °C; 15 min; Submerged up to the sternum	15 min PR	CMJ	24;48
**Yoshimura 2023**	Knee Extension Eccentric Exercise (Do minant Leg)	20.0 °C; 20 min; Immersion to the iliac crest	20 min PR	MVIC; CMJ; VAS	48
**Richards 2025**	Isometric concentric contraction of the dorsiflexors/plantarflexes of the left ankle	10.0 °C; 10 min; Soak only one calf	10 min PR	MVIC	0;24
**Wei 2025**	Lower-body eccentric exercise	11.0–15.0 °C; 12 min; Immersion to the iliac crest	12 min PR	CK	24;48;72

**Notes.**

CWI Cold Water Immersion group CON Control group MVIC Maximum Voluntary Isometric Contraction CMJ Countermovement Jump CK Creatine Kinase VAS Visual Analog Scale 0 Immediately post-intervention 24 24 h post-exercise 48 48 h post-exercise 72 72 h post-exercise PR The control group underwent seated passive recovery HIIT High-Intensity Interval Training

### Quality assessment of included studies

**Figure 3:**
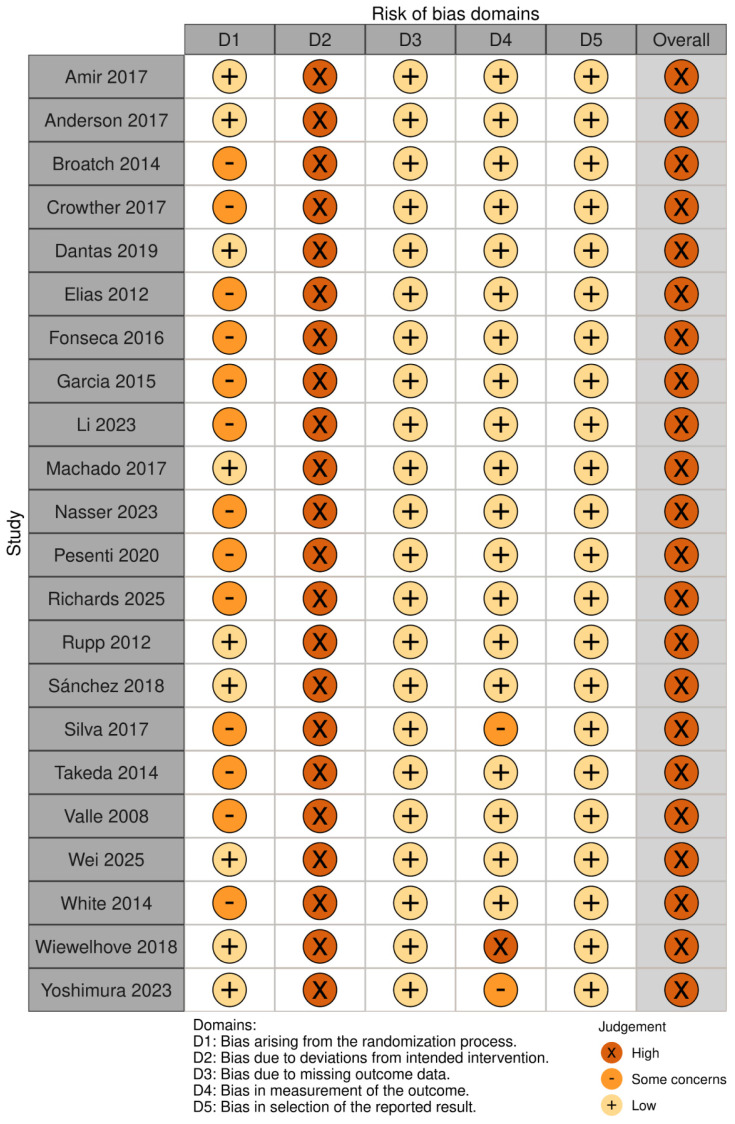
Risk of bias graph for included studies.

**Figure 4:**
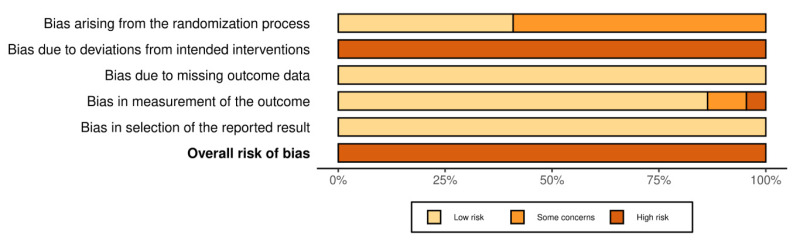
Summary of bias risk in included studies.

Based on the PEDro scale, all 22 included studies demonstrated high methodological quality (scores: 6–8/10; Table 3). Conversely, the Cochrane RoB 2 tool classified the overall risk of bias for all trials as “high” (Figs. 3 and 4). This uniform high-risk rating was exclusively driven by Domain 2 (deviations from intended interventions) due to the inherent practical impossibility of blinding participants and personnel to cold-water immersion. However, the risk of bias remained predominantly low across other key domains, including the randomization process, missing data, and reported results. Given the consistent reliance on objective physiological and biomechanical endpoints (*e.g.*, MVIC, CK) and intention-to-treat analyses, the inevitable lack of blinding is unlikely to substantially compromise the reliability of the pooled estimates.

**Table 3:** Methodological quality of the included studies assessed by the PEDro scale.

**Included studies**	**1**	**2**	**3**	**4**	**5**	**6**	**7**	**8**	**9**	**10**	**11**	**Total score**
**Amir 2017**	1	1	1	1	0	0	1	1	1	1	1	8
**Anderson 2018**	1	1	1	1	0	0	1	1	1	1	1	8
**Broatch 2014**	1	1	0	1	0	0	1	1	1	1	1	7
**Crowther 2017**	1	1	0	1	0	0	1	1	1	1	1	7
**Dantas 2019**	1	1	1	1	0	0	1	1	1	1	1	8
**Elias 2012**	1	1	0	1	0	0	1	1	1	1	1	7
**Fonseca 2016**	1	1	0	1	0	0	1	1	1	1	1	7
**Garcia 2016**	1	1	0	1	0	0	1	1	1	1	1	7
**Li 2023**	1	1	0	1	0	0	1	1	1	1	1	7
**Machado 2016**	1	1	1	1	0	0	1	1	1	1	1	8
**Nasser 2023**	1	1	0	1	0	0	1	1	1	1	1	7
**Pesenti 2020**	1	1	0	1	0	0	1	1	1	1	1	7
**Richards 2025**	1	1	0	1	0	0	1	1	1	1	1	7
**Rupp 2012**	1	1	1	0	0	1	1	1	1	1	1	8
**Sánchez 2018**	1	1	1	1	0	0	1	1	1	1	1	8
**Silva 2018**	1	1	0	1	0	0	0	1	1	1	1	6
**Takeda 2014**	1	1	0	1	0	0	1	1	1	1	1	7
**Vaile 2007**	1	1	0	1	0	0	1	1	1	1	1	7
**Wei 2025**	1	1	1	1	0	0	1	1	1	1	1	8
**White 2014**	1	1	0	1	0	0	1	1	1	1	1	7
**Wiewelhove 2018**	1	1	0	1	0	0	0	1	1	1	1	6
**Yoshimura 2023**	1	1	1	1	0	0	0	1	1	1	1	7

**Notes.**

1, subject eligibility; 2, random allocation; 3, allocation concealment; 4, baseline similarity; 5, subject blinding; 6, clinician blinding; 7, assessor blinding; 8, dropout rate <15%; 9, intention-to-treat analysis; 10, between-group statistical analysis; 11, point measurements and measures of variability

According to PEDro scale guidelines, Item 1 relates to external validity and is not used to calculate the PEDro score. The Total Score (maximum 10) is the sum of Items 2 through 11.

### Meta-analysis results

#### Maximal voluntary isometric contraction

**Figure 5:**
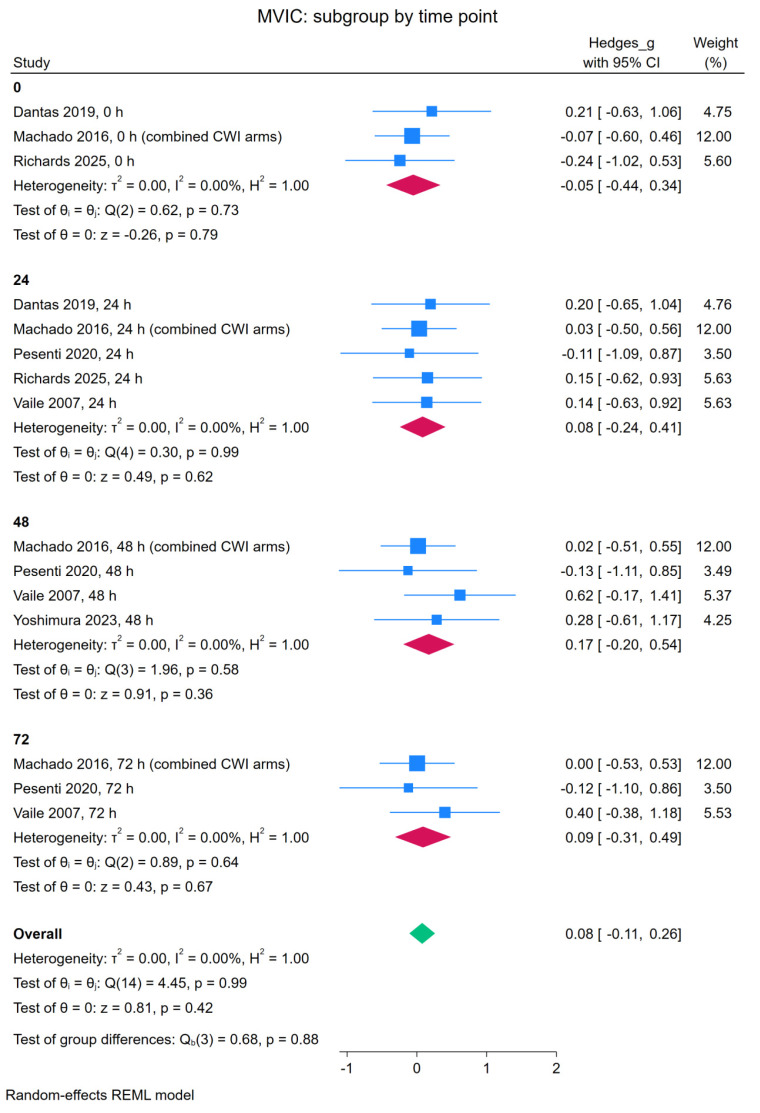
Meta-analysis forest plot of acute CWI effects on MVIC at 0 h–72 h.

Six studies contributing 15 effect sizes after multi-arm combination evaluated the effects of acute CWI on MVIC recovery over 0–72 h post-exercise (Vaile et al., 2007; Machado et al., 2016; Dantas et al., 2019; Pesenti et al., 2020; Yoshimura et al., 2023; Richards et al., 2025) (Fig. 5). The pooled analysis showed no significant effect of acute CWI *versus* control on MVIC recovery, yielding a negligible effect (g = 0.08, 95% CI [−0.11–0.26]; *z* = 0.81, *p* = 0.42), with no evidence of heterogeneity (*I*^2^ = 0.00%; Q(14) = 4.45, *p* = 0.99). Subgroup analyses consistently showed negligible and non-significant effects at 0 h (g = −0.05, 95% CI [−0.44–0.34]; *p* = 0.79), 24 h (g =0.08, 95% CI [−0.24–0.41]; *p* = 0.62), 48 h (g =0.17, 95% CI [−0.20–0.54]; *p* = 0.36), and 72 h (g =0.09, 95% CI [−0.31–0.49]; *p* = 0.67). The test for subgroup differences was not significant (Qb(3) = 0.68, *p* = 0.88), indicating that time did not moderate the effect of acute CWI on MVIC recovery.

#### Countermovement jump

**Figure 6:**
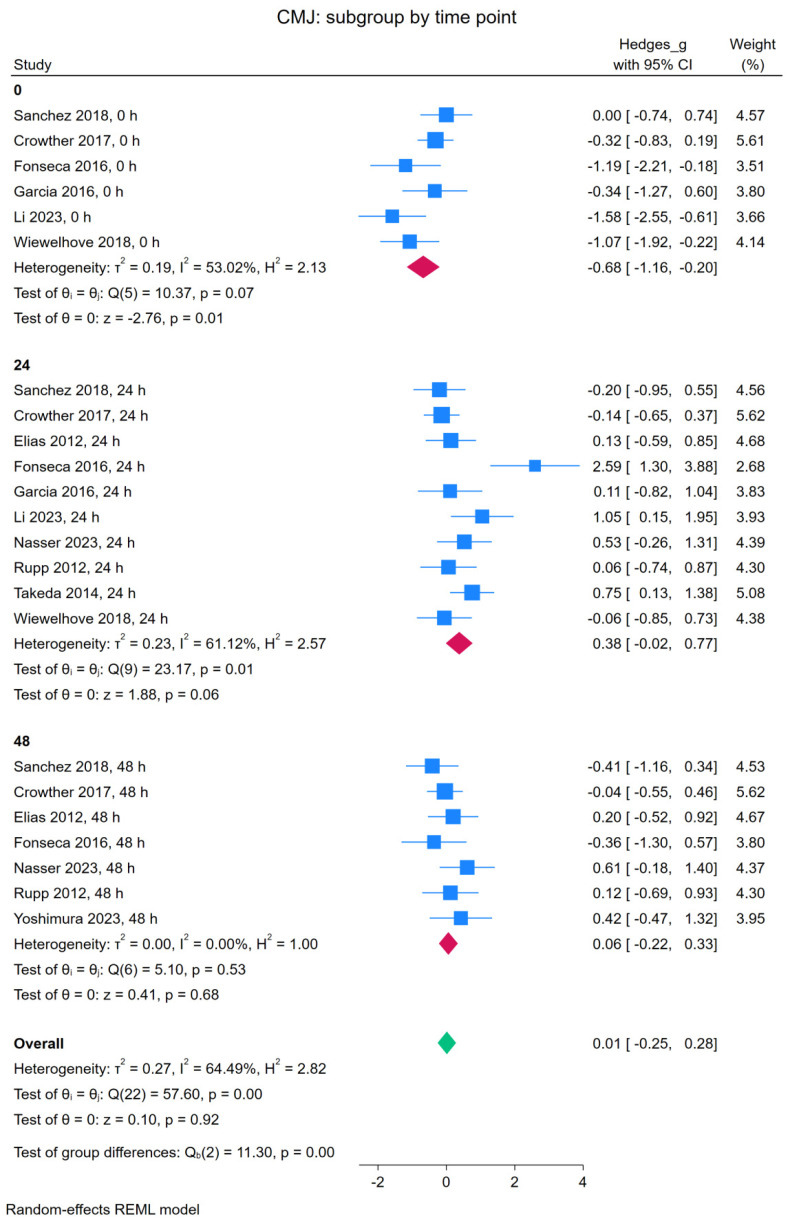
Meta-analysis forest plot of acute CWI effects on CMJ at 0 h–48 h.

Eleven studies contributing 23 effect sizes examined CMJ performance over 0–48 h post-exercise (Sánchez-Ureña, Rojas-Valverde & Gutiérrez-Vargas, 2018; Crowther et al., 2017; Elias et al., 2012; Fonseca et al., 2016; Garcia, Da Mota & Marocolo, 2016; Li et al., 2023; Nasser et al., 2023; Rupp et al., 2012; Wiewelhove et al., 2018; Yoshimura et al., 2023; Takeda et al., 2014) (Fig. 6). The overall pooled effect was not significant, indicating a negligible effect (g =0.01, 95% CI [−0.25–0.28]; *z* = 0.10, *p* = 0.92), with substantial heterogeneity (*I*^2^ = 64.49%; Q(22) = 57.60, *p* < 0.001). However, the subgroup-difference test was significant (Qb(2) = 11.30, *p* = 0.004), indicating a time-dependent effect. Specifically, acute CWI was associated with a significant, medium impairment in CMJ recovery at 0 h (g = −0.68, 95% CI [−1.16 to −0.20]; *p* = 0.01). This shifted to a small-to-moderate, non-significant positive effect at 24 h (g =0.38, 95% CI [−0.02–0.77]; *p* = 0.06), and returned to a negligible effect at 48 h (*g* = 0.06, 95% CI [−0.22–0.33]; *p* = 0.68).

#### Creatine kinase

**Figure 7:**
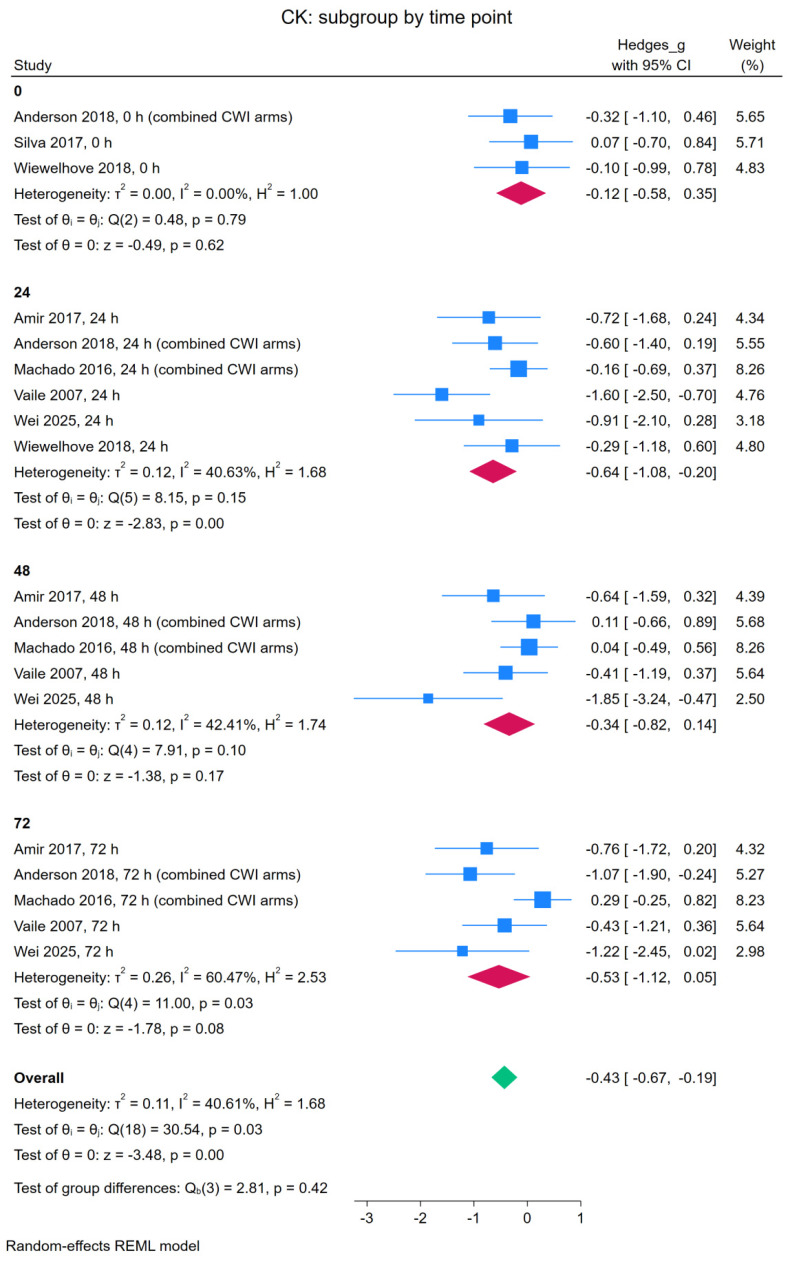
Meta-analysis forest plot of acute CWI effects on CK at 0 h–72 h.

Seven studies contributing 19 effect sizes after multi-arm combination evaluated post-exercise CK levels over 0–72 h (Amir, Hashim & Saha, 2017; Anderson, Nunn & Tyler, 2018; Machado et al., 2016; Silva et al., 2018; Vaile et al., 2007; Wei, Liu & Wang, 2025; Wiewelhove et al., 2018) (Fig. 7). The pooled analysis showed that acute CWI significantly reduced CK relative to control with a small effect (g = −0.43, 95% CI [−0.67 to −0.19]; z = −3.48, *p* < 0.001), accompanied by low-to-moderate heterogeneity (*I*^2^ = 40.61%; Q(18) = 30.54, *p* = 0.03). No significant subgroup differences were detected across time points (Qb(3) = 2.81, *p* = 0.42), suggesting no clear time-dependent effect. In the subgroup analyses, negligible-to-small, non-significant effects were observed at 0 h (g = −0.12, 95% CI [−0.58–0.35]; *p* = 0.62) and 48 h (g = −0.34, 95% CI [−0.82–0.14]; *p* = 0.17), whereas a significant, medium effect was found at 24 h (g = −0.64, 95% CI [−1.08 to −0.20]; *p* < 0.01). At 72 h, heterogeneity was substantial (*I*^2^ = 60.47%; Q(4) = 11.00, *p* = 0.03); accordingly, the random-effects model showed a non-significant medium effect (g = −0.53, 95% CI [−1.12–0.05]; *p* = 0.08).

#### Visual analog scale

**Figure 8:**
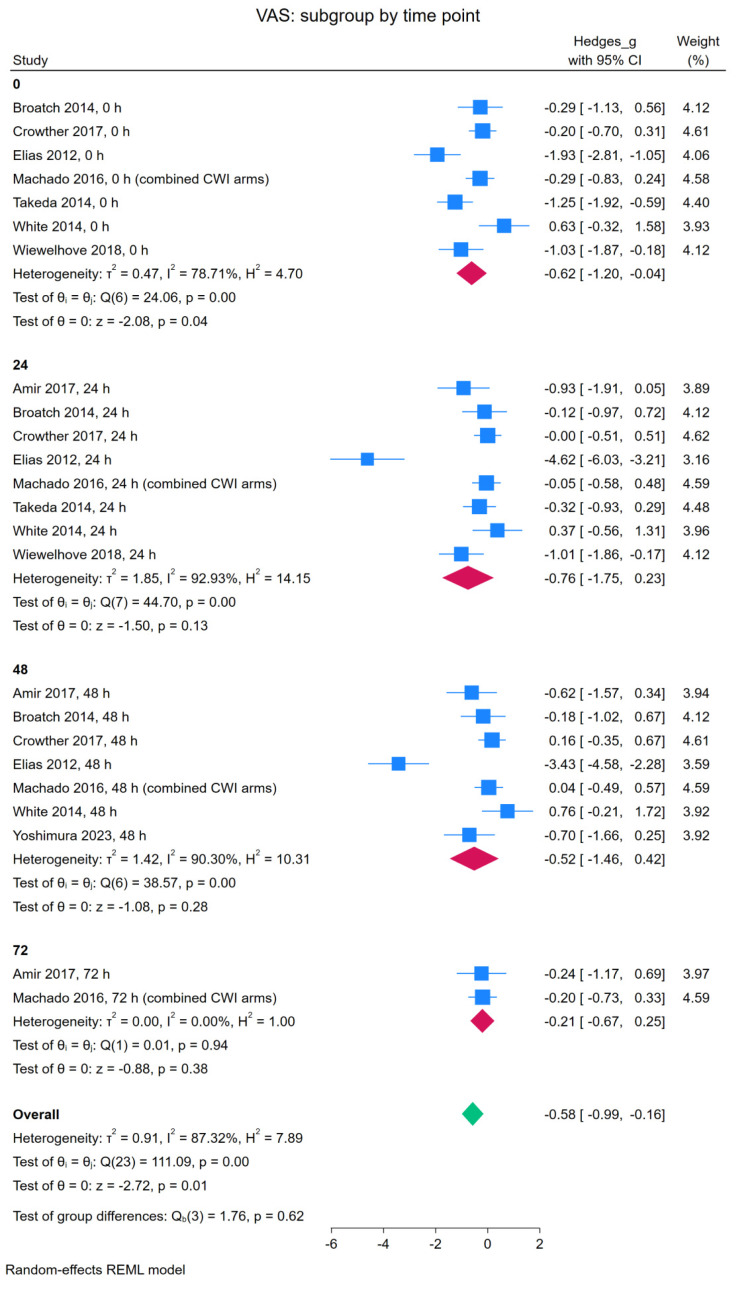
Meta-analysis forest plot of the effect of acute CWI on reducing CK levels 72 h after exercise.

Nine studies contributing 24 effect sizes after multi-arm combination examined post-exercise muscle soreness assessed by VAS over 0–72 h (Amir, Hashim & Saha, 2017; Broatch, Petersen & Bishop, 2014; Crowther et al., 2017; Elias et al., 2012; Machado et al., 2016; White, Rhind & Wells, 2014; Wiewelhove et al., 2018; Yoshimura et al., 2023; Takeda et al., 2014) (Fig. 8). The pooled analysis showed that acute CWI significantly reduced VAS scores compared with control, demonstrating a medium effect (g = −0.58, 95% CI [−0.99 to −0.16]; z = −2.72, *p* = 0.006), although heterogeneity was substantial (*I*^2^ = 87.32%; Q(23) = 111.09, *p* < 0.001). The subgroup-difference test was not significant (Qb(3) = 1.76, *p* = 0.62), indicating no statistically detectable time-dependent effect. In subgroup analyses, a significant, medium reduction in VAS was observed only at 0 h (g = −0.62, 95% CI [−1.20 to −0.04]; *p* = 0.04). The non-significant effects observed at subsequent time points corresponded to a medium effect at 24 h (g = −0.76, 95% CI [−1.75 to 0.23]; *p* = 0.13), a medium effect at 48 h (g = −0.52, 95% CI [−1.46 to 0.42]; *p* = 0.28), and a small effect at 72 h (g = −0.21, 95% CI [−0.67 to 0.25]; *p* = 0.38).

### Sensitivity analysis

**Figure 9:**
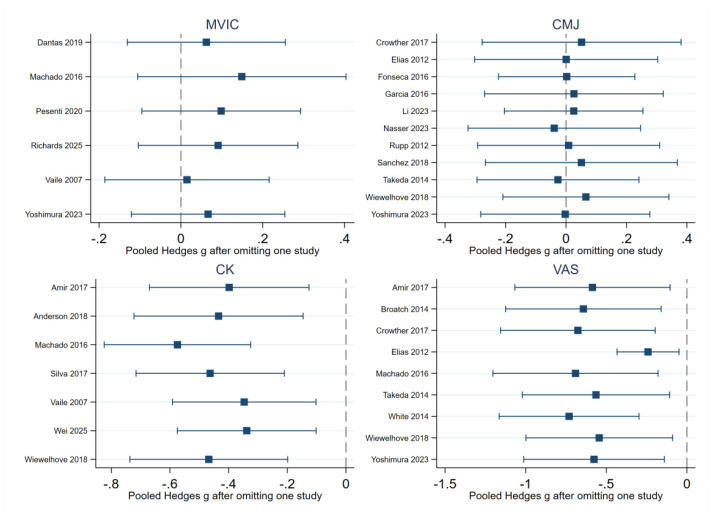
Meta-analysis forest plot of acute CWI effects on VAS at 0 h–72 h.

A leave-one-out sensitivity analysis was conducted for all primary outcomes (MVIC, CMJ, CK, and VAS) to evaluate the robustness of the pooled estimates. After multi-arm studies were combined, sequential omission of individual studies did not materially alter the direction of the pooled effects, although the statistical strength of the CK and VAS findings required cautious interpretation in subsequent sensitivity analyses (Fig. 9). A *post hoc* sensitivity analysis excluding studies that enrolled female participants did not materially change the direction of the pooled estimates, although these results should be interpreted cautiously because only a small number of studies included female participants. To account for dependency among multiple effect sizes contributed by the same study, a cluster-robust sensitivity analysis was additionally performed using study ID as the clustering variable. The null findings for MVIC (g = 0.076, 95% CI [−0.121 to 0.272]; *p* = 0.367) and CMJ (g = −0.002, 95% CI [−0.223 to 0.220]; *p* = 0.987) were unchanged. The favorable directions for CK (g = −0.348, 95% CI [−0.791 to 0.095]; *p* = 0.103) and VAS (g = −0.363, 95% CI [−0.842 to 0.115]; *p* = 0.118) were retained but were no longer statistically significant after clustering by study.

For VAS, the pooled effect remained directionally consistent after exclusion of individual studies, although the removal of Elias et al. (2012) markedly reduced heterogeneity from *I*^2^ = 87.32% to *I*^2^ = 36.20%. In addition, the extreme heterogeneity observed in the 24 h subgroup was reduced from *I*^2^ = 92.93% to 7.42%. When the analysis was recalculated using a fixed-effect model, the pooled estimate was attenuated but remained significant (g = −0.22, 95% CI [−0.37 to −0.07], *p* < 0.01).

### Publication bias

**Figure 10:**
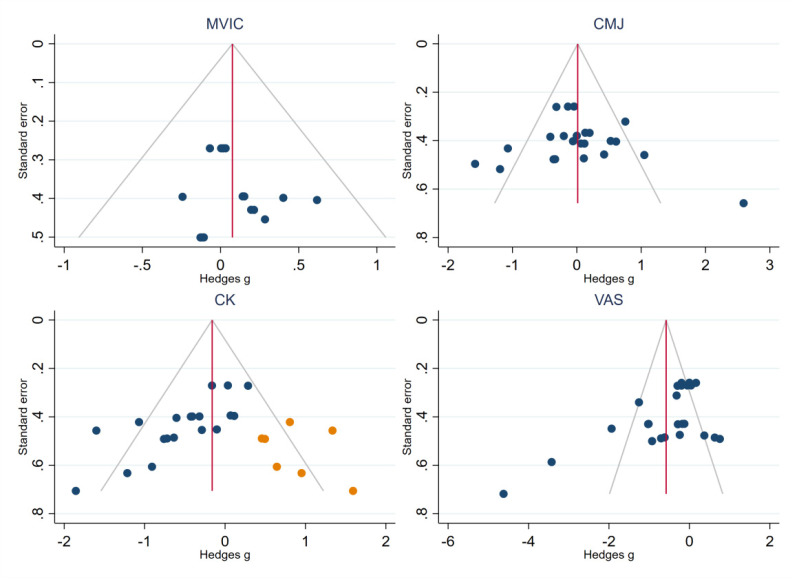
Sensitivity analysis results for MVIC, CMJ, CK, and VAS.

Publication bias was assessed using funnel plots (Fig. 10), Egger’s test, and the trim-and-fill method after multi-arm studies were combined. Visual inspection suggested approximate symmetry for MVIC and CMJ, but greater asymmetry for CK and VAS. Consistently, Egger’s test was non-significant for MVIC (*P* = 0.626) and CMJ (*P* = 0.451), but indicated potential small-study effects or publication bias for CK (*P* < 0.001) and VAS (*P* < 0.001). After trim-and-fill adjustment, the pooled CK effect was attenuated to non-significance (g = −0.160, 95% CI [−0.443–0.124]), suggesting possible overestimation of the original effect. For VAS, the trim-and-fill procedure imputed no additional studies, and the pooled estimate was unchanged (g = −0.578, 95% CI [−0.994 to −0.162]). The initial asymmetry for VAS appeared to be largely driven by a single extreme outlier (Elias et al., 2012; Elias et al., 2012), and was no longer evident after exclusion of this study (*P* = 0.471). Overall, publication bias was unlikely to have materially influenced the MVIC or CMJ findings; the CK result should be interpreted cautiously, and the VAS result should also be interpreted with caution in light of the substantial heterogeneity and outlier sensitivity.

### Evidence certainty and temporal dynamics

The certainty of evidence for the main outcomes was reassessed after multi-arm combination and is summarized in the updated Table 4. The certainty of evidence remained moderate for MVIC, low for CMJ and CK, and very low for VAS. Downgrading was primarily attributable to imprecision for MVIC, inconsistency and imprecision for CMJ, publication bias and limited information size for CK, and risk of bias, very serious inconsistency, small-study effects, and sensitivity to an outlying study for VAS.

**Table 4:** GRADE summary of findings for main outcomes.

Outcome indicators	Risk of bias	Inconsistency	Indirectness	Imprecision	Publication bias	Certainty assessment
MVIC	Not serious ↔	*I*^2^ = 0.00% ↔	Not serious ↔	Serious ↓ 95% CI crossed 0	*p* = 0.626↔	⨁⨁⨁$○ $ Moderate
CMJ	Not serious ↔	*I*^2^ = 64.49% ↓	Not serious ↔	Serious ↓ 95% CI crossed 0	*p* = 0.451↔	⨁⨁$○ $$○ $ Low
CK	Not serious ↔	*I*^2^ = 40.61% ↔	Not serious ↔	Serious ↓ Limited information size; small pooled effect	*p* < 0.001↓	⨁⨁$○ $$○ $ Low
VAS	Serious ↓	*I*^2^ = 87.32% ↓	Not serious ↔	Not serious ↔ 95% CI did not cross 0	*p* < 0.001↓	⨁$○ $$○ $$○ $ Very Low

**Notes.**

Risk of bias was not downgraded for MVIC, CMJ, and CK because these outcomes were treated as relatively objective outcomes; lack of blinding alone was not considered sufficient to lower certainty. CK is a laboratory biomarker, whereas MVIC and CMJ are standardized performance outcomes with objective recording. In contrast, VAS was downgraded by one level because it was a subjective, participant-reported outcome and therefore more susceptible to bias related to non-blinding. Imprecision was judged outcome by outcome. MVIC and CMJ were downgraded because the 95% confidence interval crossed the line of no effect. CK was conservatively downgraded because the overall information size remained limited and the pooled effect was small, despite the confidence interval not crossing 0. ↓ indicates a downgrade by one level; ↔ indicates no downgrade; *I*^2^ indicates heterogeneity.

**Figure 11:**
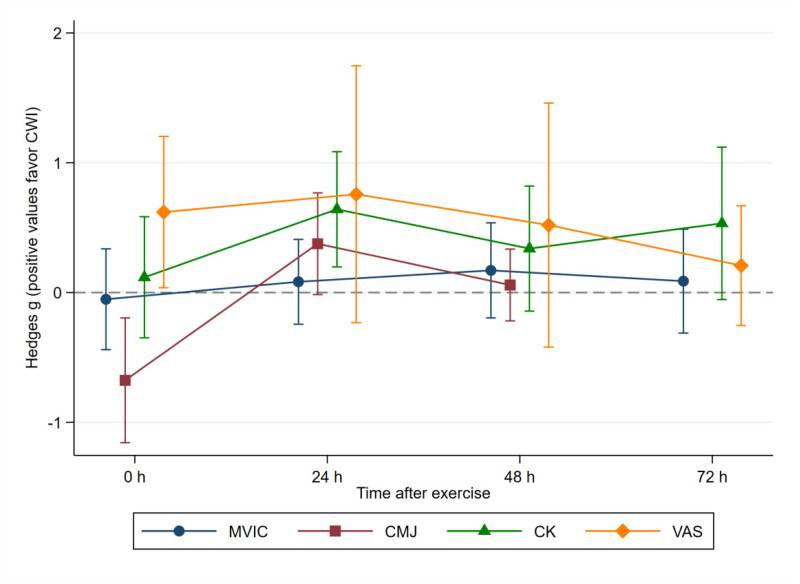
Summary of all results of the funnel plot overall overview.

To complement the outcome-specific forest plots, Fig. 11 was updated as a visual summary of the subgroup pooled effects across time points using directionally harmonized effect sizes. The updated figure showed no meaningful time-related pattern for MVIC, a transient immediate decrement in CMJ followed by attenuation at later time points, and generally favorable but imprecise patterns for CK and VAS. Figure 11 should be interpreted as a descriptive integration of subgroup pooled estimates rather than as additional inferential evidence or a continuous within-sample trajectory.

## Discussion

### Summary of evidence

In this systematic review and meta-analysis, the temporal dynamics of recovery following acute CWI were evaluated across 22 trials. For MVIC, no significant between-group differences were observed at any time point, indicating a negligible effect with no time dependence (moderate certainty). Conversely, a significant time-dependent effect was demonstrated for CMJ (low certainty); explosive performance was significantly impaired by CWI immediately post-exercise (0 h), but this between-group difference dissipated within 48 h. Furthermore, post-exercise CK levels were lower after CWI in the primary random-effects analysis; however, this effect was attenuated after trim-and-fill adjustment and was no longer statistically significant in the cluster-robust sensitivity analysis (low certainty). Finally, VAS scores were significantly reduced by CWI compared with the control condition in the primary analysis, but this finding showed substantial heterogeneity and was weakened after accounting for within-study dependency among effect sizes (very low certainty). Overall, the recovery effects of acute CWI were outcome-specific: subjective soreness tended to improve, whereas objective explosive performance (CMJ) was transiently impaired in a time-dependent manner.

### 0 h

At the immediate post-exercise assessment (0 h), CWI had no significant effect on MVIC of the affected musculature, and this neutral effect remained broadly consistent across subsequent follow-up time points. This finding was consistent with the empirical study by Lee, Moon & Kim (2025), which reported that neither whole-body nor lower-limb CWI altered the recovery trajectory of MVIC. By contrast, CWI exerted a significant immediate suppressive effect on explosive performance. This result was in line with the meta-analysis of Moore et al. (2022) which showed that dynamic or rate-dependent strength measures, such as CMJ and rate of force development (RFD), were more sensitive to CWI than static peak force. Mechanistically, the rapid decline in tissue temperature following acute cold exposure may slow nerve conduction velocity (NCV), reduce motor-unit firing frequency, and alter muscle contractile kinetics, thereby transiently impairing RFD and explosive performance (Herrera et al., 2010; Treigyte et al., 2023). Dixon et al. (2010) further demonstrated that elevating muscle temperature through a dynamic warm-up after cold exposure could partially reverse the short-term detrimental effect of CWI on CMJ, thereby providing indirect support for this temperature-dependent explanation. It should be acknowledged, however, that these mechanisms were inferred primarily from prior experimental work rather than directly measured in the studies included in the present review.

Conversely, substantial empirical evidence has shown that CWI can acutely increase pain threshold and reduce perceived muscle soreness immediately after the intervention (Machado et al., 2015; Bleakley et al., 2012). A plausible explanation is that reduced tissue temperature lowers local metabolic rate, thereby limiting interstitial oedema and tissue pressure and reducing the mechanical stimulation of nociceptors (Wang, Wang & Pan, 2025). However, this analgesic response may also be accompanied by a transient elevation in neuromuscular excitation thresholds, which could compromise high-intensity and explosive performance in the short term (Cornwall, 1994; Leeder et al., 2011). In addition, the analgesic effect of CWI may be partly attributable to cold-induced sympathetic activation, including increased catecholamine activity, which may acutely improve perceived recovery, mood, alertness, and vitality (Tabben et al., 2018; López-Ojeda & Hurley, 2024). It should also be noted that local and systemic inflammatory responses typically have not yet peaked at 0 h; therefore, any potential anti-inflammatory effects of acute CWI would be difficult to detect at this time point (Cheung, Hume & Maxwell, 2003). Taken together, the main acute features of CWI at 0 h were a neutral effect on maximal strength, a transient suppression of explosive performance, and an immediate reduction in perceived soreness. From an applied perspective, the present findings do not support the routine immediate use of CWI when another sprint-, jump-, or other power-dependent task is scheduled within a few hours. Rather, its immediate application appears more justifiable after the final training session or competition of the day, when symptom relief is prioritised over the rapid restoration of explosive performance.

### 24 h

At 24 h post-exercise, the initial suppression of explosive performance (CMJ) dissipated, indicating neuromuscular recovery. Concurrently, a significant reduction in CK levels was noted within this subgroup (g = −0.64). Mechanistically, this reduced CK efflux is attributed to cold-induced decreases in sarcolemmal permeability, hydrostatic pressure limiting cellular swelling, and subsequent reactive hyperemia facilitating waste clearance (White & Wells, 2013; Amir, Hashim & Saha, 2017). However, because the omnibus test for subgroup differences was non-significant (*P* = 0.42), this pattern does not constitute a formal statistical time-dependent effect. Furthermore, CK is an indirect and highly variable biomarker (Baird et al., 2012); its transient reduction does not definitively equate to enhanced functional recovery (Warren, Lowe & Armstrong, 1999), as controlled tissue stress and moderate inflammatory responses are critical initiators of long-term structural adaptation (Peake et al., 2017).

Similarly, perceived soreness (VAS) was mitigated at 24 h, though extreme initial heterogeneity (*I*^2^ = 92.93%) was observed. Sensitivity analysis revealed this was predominantly driven by a single outlier (Elias et al., 2012); its exclusion drastically reduced *I*^2^ to 7.42%. Because small subgroup sizes (n < 10) preclude formal meta-regression, this variance indicates profound context dependency. Specifically, the analgesic efficacy of CWI is highly sensitive to the exercise modality. As highlighted by the classic meta-analysis by Leeder et al. (2011), CWI consistently alleviates DOMS following high-intensity, metabolically demanding intermittent exercise. Conversely, its analgesic effects are substantially blunted or highly variable following heavy eccentric protocols that induce severe mechanical micro-trauma. Furthermore, deviations in immersion dose contribute to this variance. Studies by Hohenauer et al. (2015) and Wang, Wang & Pan (2025) both indicate that optimal analgesic outcomes typically require specific protocols (*e.g.*, 10–15 °C for 10–15 min), and deviations from this precise dosage range can significantly alter the magnitude of perceived recovery. Crucially, subjective VAS is highly susceptible to expectancy bias. Recent placebo-controlled trials suggest that soreness alleviation is at least partially driven by psychological effects (Nasser et al., 2023). Moreover, highly trained athletes typically exhibit blunted perceptual responses to muscle damage due to the “repeated bout effect” (Howatson et al., 2009), which may dilute the apparent subjective benefits of CWI compared to recreational populations.

Therefore, the overall recovery outcomes observed at 24 h—encompassing both objective CK reductions and subjective VAS mitigation—should not be directly equated with substantial structural repair of muscle tissue. Rather, they reflect a complex interaction among specific exercise stressors, cooling dosages, individual physiological adaptations, and psychological expectancy.

### 48–72 h

By 48 to 72 h post-exercise, the pooled effects of acute CWI on functional measures (MVIC and CMJ) decayed to near-baseline levels. Although CK and VAS still trended in favor of CWI, neither reached statistical significance, indicating that the marginal benefits of a single bout of cold-water immersion are minimal during the late recovery phase. Overall, this pattern suggests that as the recovery process enters the broader damage–inflammation–remodeling continuum, the subsequent strength recovery trajectory is predominantly dictated by the nature of the original exercise stimulus and the EIMD itself, rather than being continuously governed by a single, brief cold exposure (Markus et al., 2021; Moore et al., 2022). Notably, the surge in heterogeneity within the 72 h CK subgroup (*I*^2^ = 60.47%) is primarily attributable to the intervention modalities, as this subgroup predominantly utilized heavy eccentric resistance protocols (*e.g.*, Machado et al., 2016: 75% one-rep max (1RM), five sets of eccentric resistance training (Machado et al., 2016; Wei, Liu & Wang, 2025: lower-body eccentric exercise (Wei, Liu & Wang, 2025)). These mechanically demanding, eccentric-heavy loads induce severe structural micro-trauma, which typically results in delayed CK efflux kinetics (often peaking at 48–96 h). Furthermore, as an indirect and highly variable biomarker, CK responses are modulated not only by recruited muscle mass and individual biological differences but also by specific CWI dosages (temperature and duration) (Ahokas et al., 2020). Therefore, the high heterogeneity and the crossing-zero confidence interval at 72 h are more logically explained by the complex interplay among extreme exercise models, CWI dose discrepancies, and the inherent delayed, highly variable kinetics of CK, rather than a stable, reproducible advantage of CWI in accelerating late-stage clearance.

Given the physiological limitations of cold exposure during the late remodeling phase, CWI is not inherently superior to alternative thermal strategies. Recent meta-analytical evidence demonstrates that contrast water therapy (CWT) ranks higher for accelerating CK clearance, whereas CWI exhibits superior efficacy for DOMS alleviation, highlighting distinct, outcome-specific benefits (Higgins, Greene & Baker, 2017). Furthermore, empirical trials reveal that while CWI and hot water immersion (HWI) elicit different acute physiological responses, neither consistently outperforms passive recovery between 24 and 72 h post-exercise (Wellauer et al., 2025). Crucially, practitioners must strictly differentiate acute recovery from long-term adaptation. Existing meta-analyses strongly warn that the routine application of CWI following resistance training blunts anabolic signaling, thereby attenuating muscle hypertrophy and strength gains (Malta et al., 2020). Consequently, the transient short-term benefits of acute CWI should not be conflated with positive structural adaptations in long-term training cycles.

### Research limitations

Several limitations inherent in the present review warrant cautious interpretation. First, a profound sex imbalance across the included studies (362 males *vs.* 13 females) severely restricts the generalizability of these findings. Given the established sex-based differences in thermoregulatory responses and inflammatory kinetics, the present pooled effects cannot be reliably extrapolated to female athletes. Second, the inherent inability to blind participants to the CWI intervention inevitably introduces expectancy bias. This likely inflates subjective outcomes such as VAS, which was consequently rated as having a very low certainty of evidence. Third, several included trials possessed very small sample sizes (minimum *n* = 7 per arm). Although leave-one-out sensitivity analyses confirmed the overall directional stability of the pooled estimates, Egger’s test and trim-and-fill adjustments revealed significant publication bias and small-study effects. Notably, the pooled CK effect was rendered non-significant after trim-and-fill adjustment, suggesting the original small benefit was likely overestimated. Fourth, several included studies contributed multiple effect sizes across time points, and two trials included multiple CWI arms sharing a common control group. We addressed this by combining multi-arm comparisons before effect-size calculation and by adding a cluster-robust sensitivity analysis by study ID; however, the limited number of study clusters means that this analysis should be interpreted as supportive rather than definitive. Fifth, considerable heterogeneity existed regarding specific CWI dosages (*e.g.*, water temperature, immersion depth, and duration) and exercise modalities. Because the limited number of trials precluded formal meta-regression or dose-stratified subgroup analyses (*e.g.*, evaluating temperatures ≤ 10 °C *vs.* 10–15 °C), the optimal parameters for CWI application remain loosely defined. Finally, this review evaluated only single, acute CWI exposures over a short-term follow-up (≤72 h), offering no insight into the potential blunting effects of repeated cold exposure on long-term structural adaptations. Future well-powered RCTs must explicitly investigate sex-specific responses, standardize dose–response parameters, and integrate both acute recovery and long-term adaptation metrics.

## Conclusion

This systematic review and meta-analysis indicates that acute CWI elicits outcome-specific rather than uniformly time-dependent effects during post-exercise recovery. CWI did not significantly enhance MVIC recovery and transiently impaired CMJ immediately after exercise, although this decrement dissipated within 48 h. CWI was associated with lower CK and VAS values in the primary random-effects analyses, but these findings should be interpreted cautiously because they were sensitive to publication-bias, heterogeneity, and dependency-adjusted sensitivity analyses. From an applied perspective, acute CWI may be most appropriate when short-term symptom relief is prioritized after congested scheduling, but it should be avoided immediately before tasks requiring maximal power output. Future well-powered, sex-balanced trials are required to standardize optimal dosages and clarify the balance between acute recovery and long-term adaptation.

## Supplemental Information

10.7717/peerj.21537/supp-1 Supplemental Information 1 PRISMA checklist.

10.7717/peerj.21537/supp-2 Supplemental Information 2 Search queries, supplemental figures, and supplemental tables

10.7717/peerj.21537/supp-3 Supplemental Information 3 Raw data.

## Additional Information and Declarations

### Data Availability

The following information was supplied regarding data availability: The raw data is available in the Supplemental Files.
