# Supplementary material for: Temporal dynamics of muscle strength recovery following acute cold-water immersion: a systematic review and meta-analysis
Source: PeerJ. 2026 Jul 16;14:e21537. doi: 10.7717/peerj.21537 (PMC13380888; doi:10.7717/peerj.21537)

Temporal Dynamics of Muscle Strength Recovery Following Acute Cold-Water Immersion: A Systematic Review and Meta-Analysis

# Database search queries

## Cochrane library


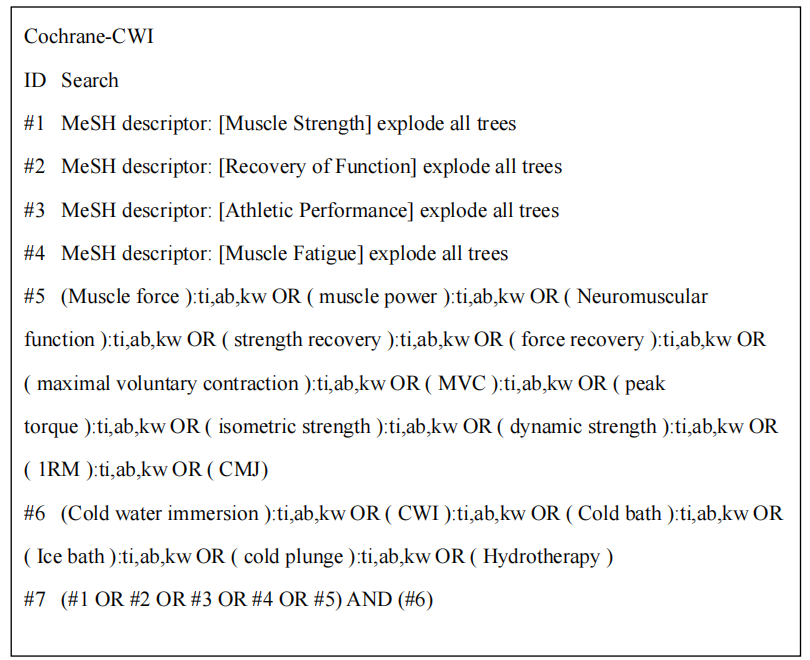


## Embase


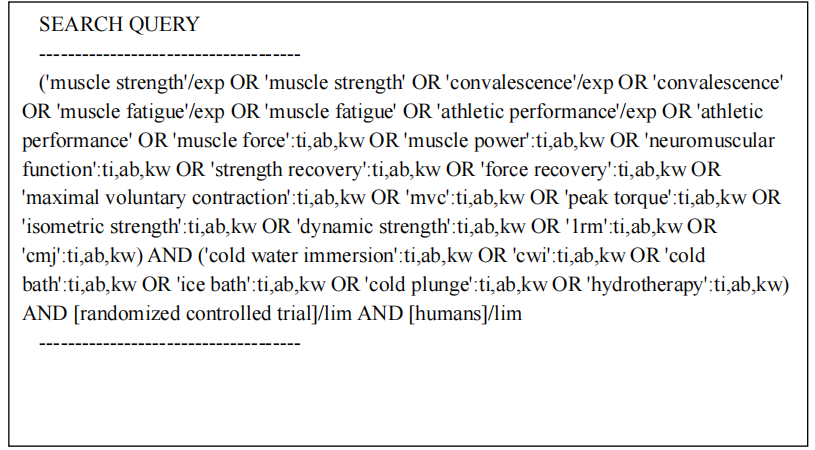


## Web of Science


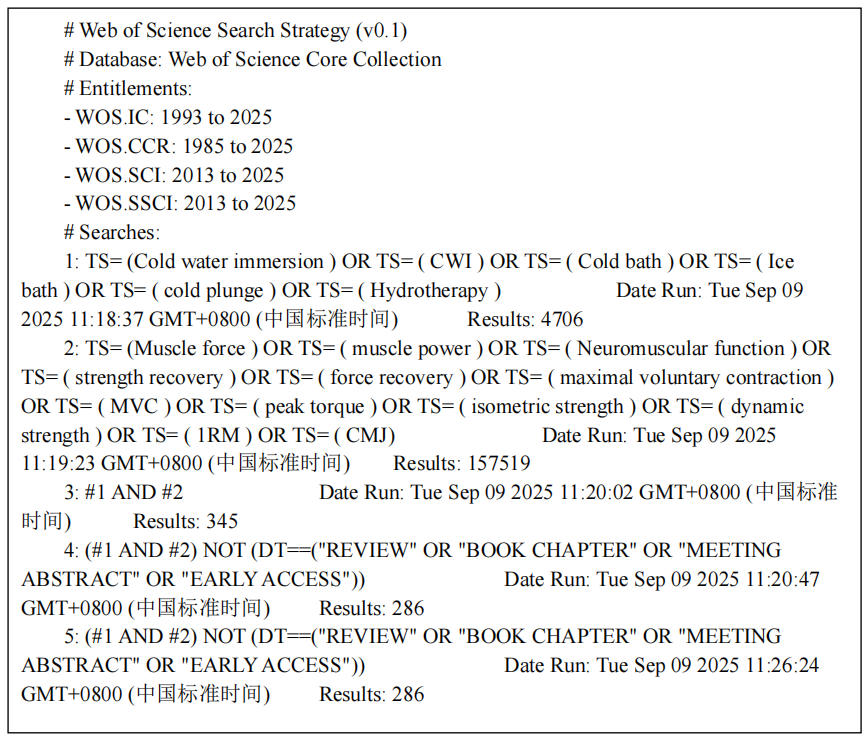


# Funnel chart for subgroups by time period

## MVIC


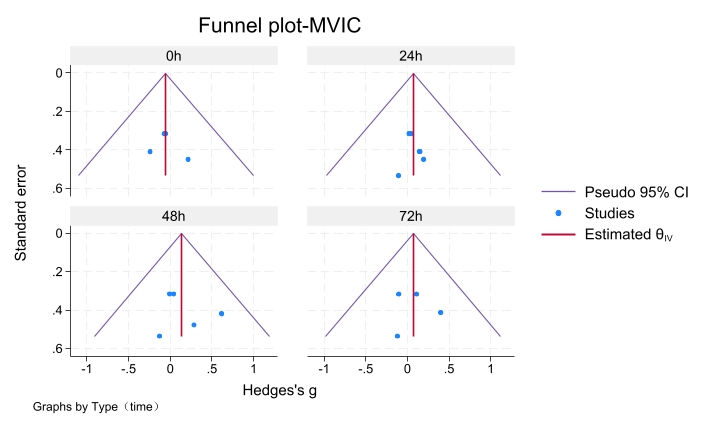


## CMJ


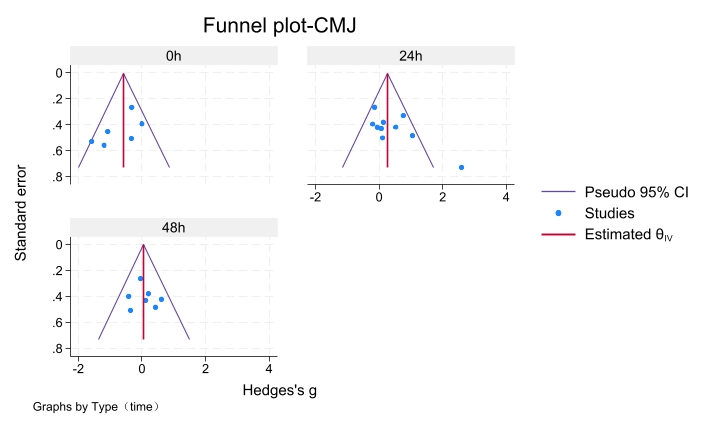


## CK


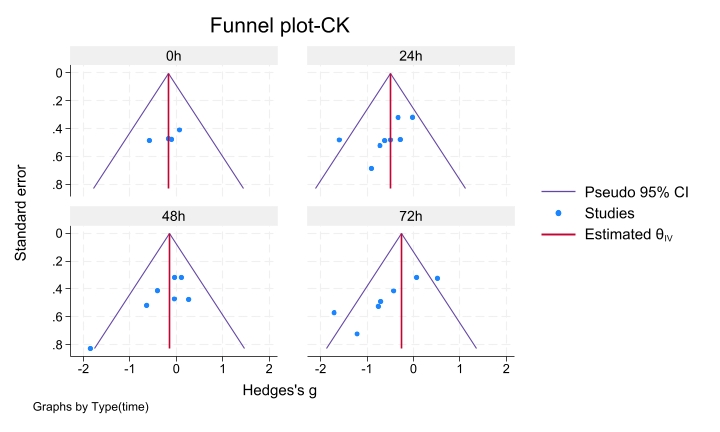


## VAS


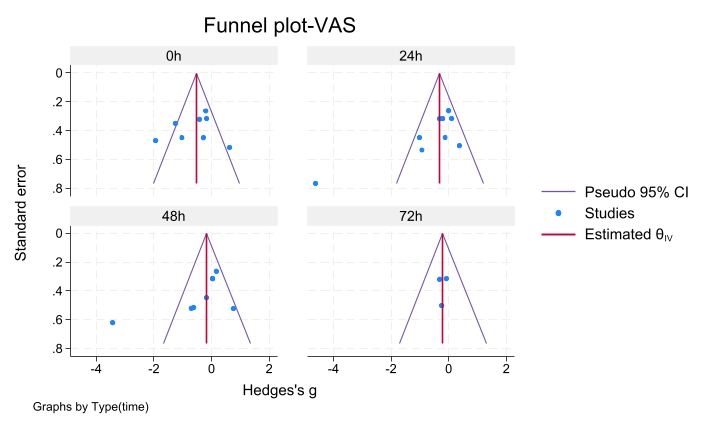


# Dependency-adjusted sensitivity analysis after combining multi-arm studies

| outcome | effect_sizes | study_clusters | overall_result | overall_p | direction | interpretation |
| --- | --- | --- | --- | --- | --- | --- |
| MVIC | 15 | 6 | 0.076 [-0.121, 0.272] | 0.367 | Positive values favor CWI | Stable null finding after clustering by study. |
| CMJ | 23 | 11 | -0.002 [-0.223, 0.220] | 0.987 | Positive values favor CWI | Overall null finding remained |
| CK | 19 | 7 | -0.348 [-0.791, 0.095] | 0.103 | Negative values favor CWI | Favorable direction, but no longer statistically significant after clustering. |
| VAS | 24 | 9 | -0.363 [-0.842, 0.115] | 0.118 | Negative values favor CWI | Favorable direction, but no longer statistically significant after clustering. |

# Forest plot of changes in pain intensity (VAS) across different follow-up time points (excluding the study by Elias et al., 2012)


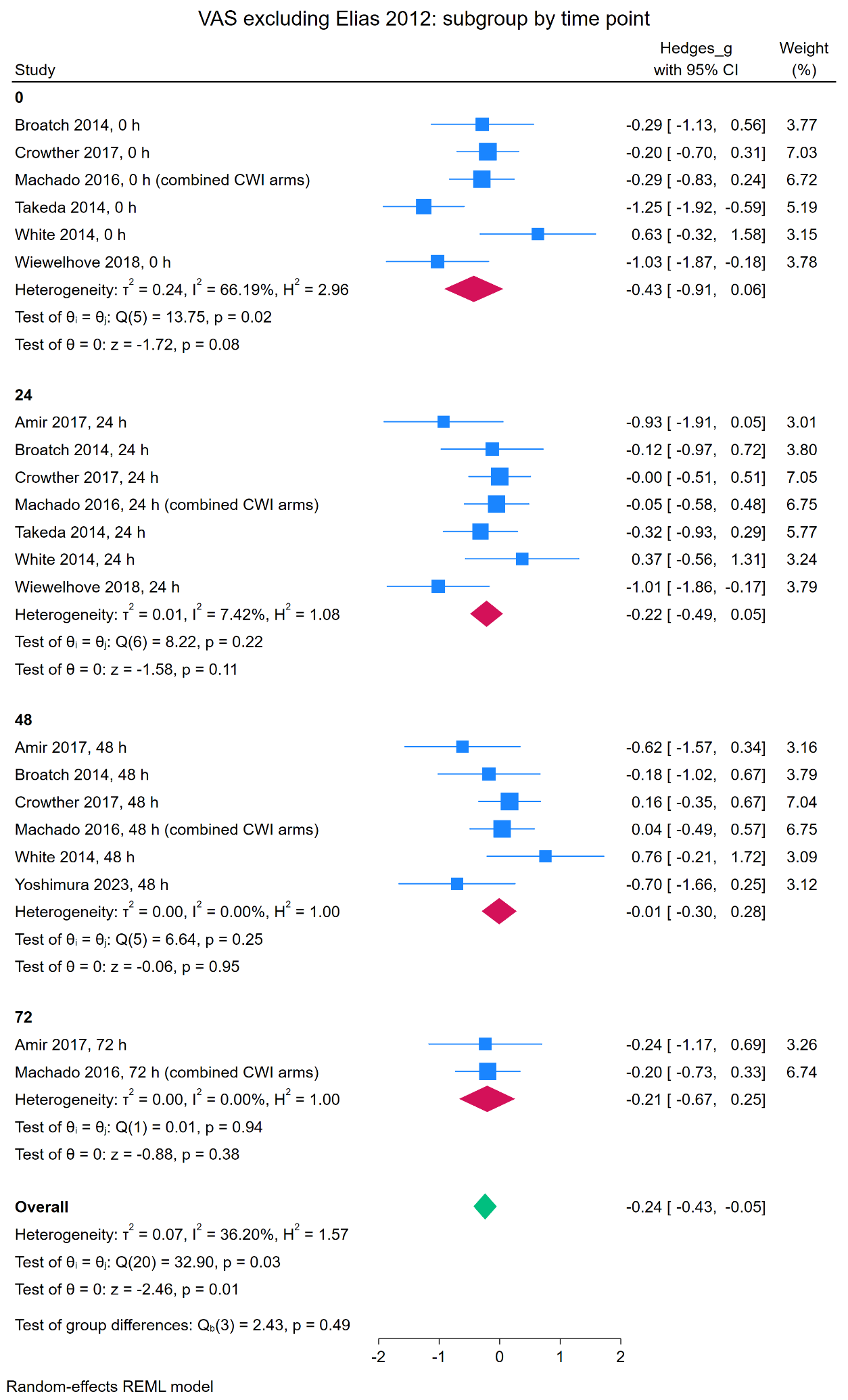

Supplement: Supplemental Information 2 [file peerj-14-21537-s002.docx]
